# The roles of autophagy and mitophagy in corneal pathology: current knowledge and future perspectives

**DOI:** 10.3389/fmed.2023.1064938

**Published:** 2023-04-21

**Authors:** Rajalakshmy Ayilam Ramachandran, Jose Marcos Sanches, Danielle M. Robertson

**Affiliations:** Department of Ophthalmology, The University of Texas Southwestern Medical Center, Dallas, TX, United States

**Keywords:** cornea, autophagy, mitophagy, mitochondria, epithelium, endothelium, stroma

## Abstract

The cornea is the clear dome that covers the front portion of the globe. The primary functions of the cornea are to promote the refraction of light and to protect the eye from invading pathogens, both of which are essential for the preservation of vision. Homeostasis of each cellular layer of the cornea requires the orchestration of multiple processes, including the ability to respond to stress. One mechanism whereby cells respond to stress is autophagy, or the process of “self-eating.” Autophagy functions to clear damaged proteins and organelles. During nutrient deprivation, amino acids released from protein breakdown *via* autophagy are used as a fuel source. Mitophagy, a selective form of autophagy, functions to clear damaged mitochondria. Thus, autophagy and mitophagy are important intracellular degradative processes that sustain tissue homeostasis. Importantly, the inhibition or excessive activation of these processes result in deleterious effects on the cell. In the eye, impairment or inhibition of these mechanisms have been associated with corneal disease, degenerations, and dystrophies. This review summarizes the current body of knowledge on autophagy and mitophagy at all layers in the cornea in both non-infectious and infectious corneal disease, dystrophies, and degenerations. It further highlights the critical gaps in our understanding of mitochondrial dysfunction, with implications for novel therapeutics in clinical practice.

## 1. Introduction

The cornea is a five layered tissue that functions to refract light as it enters the eye and to protect the eye from invasion by pathogens (1). Of the five layers, Bowman’s layer is a modified basement membrane just beneath the corneal epithelium, while Descemet’s membrane is posterior to the stroma, functioning as a basement membrane for the corneal endothelium. The anterior-most cell layer of the cornea is composed of a five to seven cell layered stratified epithelium in various stages of differentiation. This includes basal cells that are able to undergo mitosis, wing or intermediate cells, and squamous cells that are contiguous with the conjunctival epithelium, forming the ocular surface. The next cellular layer is the collagenous stroma, the thickest layer of the cornea. The stroma is composed of predominantly type I collagen fibrils. It is the organization of these fibrils that contribute to corneal strength and transparency. The collagen is maintained by keratocytes localized within the corneal stroma. These cells have essential roles in corneal wound healing and fibrosis. Finally, the most posterior cellular layer in the cornea is the endothelium. This monolayer of cells functions to maintain corneal hydration. In humans, the corneal endothelium does not undergo cell division, but instead exhibits gross morphological changes following cell loss.

Autophagy or macroautophagy is a cannibalistic process whereby the cell “eats” misfolded proteins and damaged organelles in order to maintain homeostasis and generate energy for the cell (2). During the initial stages of autophagy, the isolation membrane or phagophore forms around cytoplasmic constituents that are destined for degradation, known as cargo. This double membrane structure continues to elongate until closing, encapsulating the cargo within the mature autophagosome. The autophagosome then docks and undergoes subsequent fusion with the lysosome where the cargo is subject to proteolysis by hydrolases. These raw materials are then recycled into macromolecules that are used in energy homeostasis. The microtubule-associated protein I light chain (LC3-I) is an integral protein involved in autophagy. LC3-I is an autophagosomal protein that is present within the inner and outer membranes. During autophagy, LC3-I undergoes lipidation to LC3-II. This shifts LC3 from the cytoplasm to the autophagosomal membrane during elongation, where it plays a key role in the conjugation of other autophagy proteins.

**FIGURE 1:**
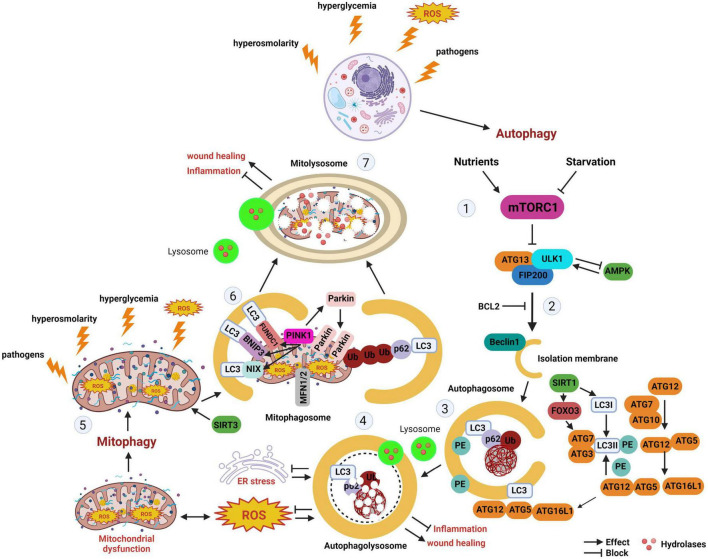
Autophagy and mitophagy. The stages of autophagy and mitophagy, a selective form of autophagy that clears damaged mitochondria. (Stage 1) Autophagy induction. Nutrient starvation and hyperglycemia differentially regulate mTORC1. During nutrient starvation, AMPK activates ULK1 to induce autophagy. In hyperglycemia, AMPK and SIRT1 inhibits autophagy. (Stage 2) Autophagy nucleation. Beclin 1 is liberated from BCL2, initiating autophagosome nucleation. (Stage 3) Autophagy expansion/completion. LC3II is formed from the association of LC3 with PE. SIRT1 interacts with autophagy proteins ATG5, ATG7, and LC3, as well as FOXO3. The autophagosome undergoes fusion with the lysosome. (Stage 4) Autophagy termination. Mature lysosomes are fused forming autophagolysosomes. Cargo undergoes proteolytic degradation by hydrolases. (Stage 5) Hyperglycemia, hypoxia, ROS, and other factors may activate mitophagy. (Stage 6) Depolarized mitochondria promote the recruitment of PINK1 to the mitochondria. PINK1 accumulation at the outer mitochondrial membrane leads to the recruitment of Parkin. As an E-3 ligase, Parkin promotes polyubiquitination of other proteins, including p62, leading to recognition of LC3 and mitophagosome formation. BNIP3, BNIP3L/NIX, and FUNDC1 are mitophagy receptors with LIR that can directly interact with LC3 on the autophagosome in the absence of PINK1 accumulation. (Stage 7) Like autophagy, mitophagosomes fuse with lysosomes, allowing for damaged mitochondria to undergo degradation by lysosomal enzymes. mTORC1, mechanistic target of rapamycin complex 1; ATG, autophagy related protein; ULK1, Unc-51-like kinase 1; FIP200, focal adhesion kinase family interacting protein; AMPK, adenosine monophosphate-activated protein kinase; SIRT1, sirtuin-silent mating type information regulation 2 homolog 1; SIRT3, sirtuin-silent mating type information regulation 2 homolog 3; BCL2, B-cell lymphoma; FOXO3, forkhead box O3; LC3, microtubule-associated protein 1A/1B-light chain 3; Ub, ubiquitin; p62, also known as p62/SQSTM1 (sequestosome 1); PE, phosphatidyl ethanolamine; ER, endoplasmic reticulum; ROS, reactive oxygen species; BNIP3, BCL2/adenovirus E1B interacting protein 3; BNIP3L/NIX, pro-apoptotic protein related to the BH3-only family; MFN1/2, mitofusin 1/2; PINK1, PTEN-induced kinase 1; FUNDC1, FUN14 domain containing 1; ATP, adenosine triphosphate.

Mitophagy is a form of selective autophagy that degrades and recycles damaged mitochondria (3). There are multiple pathways through which mitophagy proceeds. In the presence of mitochondrial damage that results in mitochondrial depolarization, presenilins-associated rhomboid-like protein (PARL) activity is impaired, leading to stabilization of the PTEN induced kinase 1 (PINK1) at the outer mitochondrial membrane. This triggers recruitment of the E3 ligase PARKIN, which then ubiquitinates additional mitochondrial membrane proteins, targeting them for proteasomal degradation or the autophagy adapter p62. P62 in turn binds to LC3-II on the autophagosome. In non-PINK1 mediated mitophagy, mitophagy receptors that express LC3-II interacting region (LIR) are able to directly recruit LC3-II labeled autophagosomes. A schematic of the major autophagy and mitophagy pathways is detailed in Figure 1.

While autophagy is a heavily conserved mechanism, mitophagy differs somewhat in mammals. Moreover, the role of autophagy and mitophagy in human disease appears to be cell and context specific. In this review, we summarized the current state of knowledge regarding autophagy and mitophagy in non-infectious and infectious corneal disease. We further highlight the major gaps in knowledge that exist with respect to these pathways in the cornea.

## 2. Non-infectious corneal diseases, dystrophies, and degenerations

### 2.1. The diabetic cornea

**FIGURE 2:**
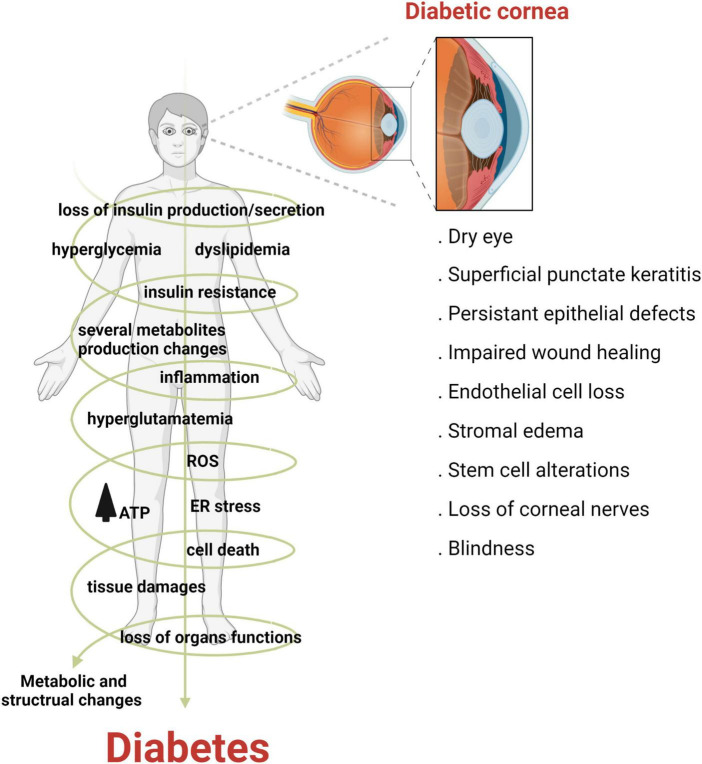
Diabetes adversely effects the cornea. Diabetes is a multifactorial disease characterized by metabolic dysfunction throughout the human body. In the cornea, diabetes can cause a host of complications ranging from dry eye disease to blindness, in severe cases.

Diabetes mellitus (DM) is a complex and multifactorial disease characterized by progressive and chronic metabolic impairment due to the loss of insulin production and secretion from beta cells in the pancreas. This results in abnormal insulin signaling and, consequently, drives hyperglycemia and dyslipidemia (4). The prevalence of diabetes is increasing worldwide. Indeed, it has been estimated that by 2045, over 600 million adults will suffer from diabetes (5). Corneal complications from diabetes are common and occur in over two thirds of the diabetic population (6). Unlike diabetic retinopathy, corneal complications are painful in the early stages of disease, with pain levels decreasing in patients with fulminant nerve loss, a condition known as corneal neuropathy (7). The spectrum of diabetic corneal complications includes dry eye disease, corneal erosions, persistent epithelial defects and ulcerations, impaired wound healing, epithelial edema, alterations in limbal stem cells and in severe cases, blindness (Figure 2) (8–10).

As described earlier, autophagy is a vital cellular pathway that involves the recycling of misfolded proteins and damaged organelles to facilitate cell and tissue homeostasis. It is well established that too little or too much autophagy contributes to the development of many diseases. This is due to the cell being unable to recycled damaged components when autophagy is inhibited; whereas, excessive autophagy can lead to autophagic cell death. In the cornea, the dysregulation of canonical macro autophagy and mitophagy have been suggested as potential contributory mechanisms associated with the pathophysiology of diabetes (11–13). However, the relationship between autophagy and mitophagy with corneal complications in diabetes has yet to be fully elucidated.

Recent work evaluated the effects of hyperglycemia on the expression of the nicotinamide adenine dinucleotide (NAD+) dependent deacetylase sirtuin, the silent mating type information regulation 2 homolog 3 (SIRT3), and proteins involved in the forkhead box O-3A (FOXO3A)/PINK1-Parkin axis (14). SIRT3 localizes to the mitochondria and regulates key metabolic functions during stress (15). Using TKE2 mouse corneal epithelial progenitor cells *in vitro* and Ins2*^Akita/+^* mice *in vivo*, Hu et al. reported a considerable decrease in SIRT3 expression, as well as LC3B, after culture in high glucose and in the diabetic cornea, suggesting the suppression of mitophagy in diabetes (14). They then used an adenoviral vector to over-express SIRT3, which led to an increase in LC3B *in vitro* and *in vivo* and augmented the FOXO3a/PINK1-Parkin pathway. This enhanced corneal epithelial cell migration in culture and promoted epithelial wound healing in the diabetic mouse cornea.

SIRT1 is a NAD-dependent deacetylase with pivotal roles in fat and glucose metabolism (16, 17). SIRT1 has been shown to modulate insulin sensitivity and apoptosis through the deacetylation of p53 (18, 19). This post-translational modification of p53 exerts a protective role in corneal wound healing *via* the insulin-like growth factor (IGF)-1/AKT pathway. The IGF pathway is dysregulated in the diabetic cornea due in part to an increase in expression of the IGF binding protein (BP)-3 in tear fluid (20–23). Wang et al. showed that hyperglycemia reduced SIRT1 expression in corneal epithelial cells in culture and in the *Ins2^Akita/+^* mouse model. The decrease in SIRT1 expression was associated with increased p53 acetylation and suppressed AKT signaling in corneal epithelial cells. In contrast, rescue of SIRT1 expression promoted cell migration. This corresponded to a reduction in acetylated p53 and expression levels of IGFBP-3, while AKT signaling was restored. While this study did not directly investigate autophagy, given the established role for IGFBP-3 in the inhibition of autophagy in corneal epithelial cells, we hypothesize that there would be an increase in autophagy in cells expressing high levels of SIRT1 (24). This would be consistent with the relationship between SIRT3 and mitophagy.

Similar to the diabetic corneal epithelium, hyperglycemia has also been shown to downregulate mitophagy in corneal endothelial cells. Using a cell culture model, human corneal endothelial cells (HCECs) grown in hyperglycemic conditions exhibited altered mitochondrial morphology and a decrease in certain mitophagy proteins including LC3B, Parkin, PINK1, and the mitophagy receptor BNIP3L/Nix (25). This was associated with a decrease in Na+/K+ ATPase and zonula occludens-1 (ZO-1). The decrease in the Na+/K+ ATPase was further associated with an increase in corneal thickness due to stromal edema in a streptozotocin-induced type 1 diabetic mouse model. Exposing HCECs to hyperglycemic conditions while co-treating with carbonyl cyanide m-chlorophenyl hydrazine (CCCP), a compound that robustly depolarizes mitochondria thereby inducing PINK-mediated mitophagy, increased LC3B and PINK1 expression and decreased reactive oxygen species (ROS) production. The upregulation of mitophagy was also associated with increased levels of Na+/K+ ATPase and ZO-1. *In vivo*, treatment with CCCP restored corneal thickness values to control levels.

While insulin has not been studied as a mediator of autophagy or mitophagy in the corneal epithelium in the setting of hyperglycemia, insulin has been shown to inhibit mitophagy in corneal epithelial cells (26). Titone et al. used telomerase-immortalized human corneal epithelial (hTCEpi) cells and primary cultured human corneal epithelial cells to investigate the effects of insulin on metabolic activity and mitochondrial quality control in cells exposed to growth factor withdrawal (26). They reported that insulin blocks PINK-mediated mitophagy by preventing mitochondrial depolarization during prolonged stress. This occurred through activation of the insulin receptor. Surprisingly, however, the authors reported on the translocation of insulin receptor, insulin-like growth factor type receptor, and epidermal growth factor receptor to the mitochondria. The ability of these mitochondrial-localized receptors to stabilize the mitochondria and mediate mitophagy remains unknown.

In addition to metabolic changes in the corneal epithelium, chronic hyperglycemia is associated with loss of the corneal subbasal nerve plexus (27–30). Together these changes cause cell and tissue damage (6, 7). Corneal nociceptors are derived from the trigeminal ganglion. Assessment of the trigeminal ganglion in hyperglycemic culture and in STZ-induced type 1 diabetic mice has shown an increase in the microRNA, miR-34c, that paralleled a reduction in the expression of key autophagy proteins, ATG4B and LC3-II (31). The authors further showed that local inhibition of miR-34c *via* a subconjunctival injection with a miR-34c antagomir, promoted corneal wound healing. Importantly, the downregulation of miR-34c was also associated with an increase in autophagy (31). A subsequent study by this same group showed similar results with miR-181a. Specifically, they found that miR-181a was also highly expressed in STZ-treated diabetic mice and in neurons cultured in high glucose *in vitro* (32). The upregulation of miR-181 similarly led to a down regulation of autophagy-related proteins ATG5, LC3B-II, and B-cell lymphoma-2 (BCL-2). Again, the inhibition of miR-181a restored autophagy, promoted the expansion of neuronal axons *in vitro*, and showed evidence of nerve repair *in vivo*. These findings, summarized in Table 1, suggest that the modulation of autophagy to increase autophagic flux may be useful in the prevention and treatment of diabetic keratopathy.

**TABLE 1:** The role of autophagy/mitophagy in corneal diseases, dystrophies, and degenerations.

Corneal disease/dys/deg*	Autophagy	Mitophagy	Phenotype/physiological significance
Diabetes/hyperglycemia	Decreased	Decreased	Delay in corneal epithelial wound healing Increases corneal opacity and corneal thickness Endothelial cell dysfunction Corneal neuropathy
Dry eye disease/hyperosmolarity/desiccating stress	Increased	Increased	Reduction of inflammation Improvement in cellular homeostasis
Limbal stem cells	Increased	Unknown	Enhancement in limbal epithelial stem cell population Cellular homeostasis Tissue development
Keratoconus	Equivocal	Decreased	Corneal thinning
Fuchs corneal endothelial dystrophy	Increased	Increased	Mitochondrial damage Endothelial cell dysfunction
Antioxidant defenses in corneal disease	Increased	Unknown	Possible linkage between antioxidants and autophagy *via* NRF2

*Corneal disease/dys/deg, corneal disease/dystrophies/degenerations.

### 2.2. Dry eye disease

**FIGURE 3:**
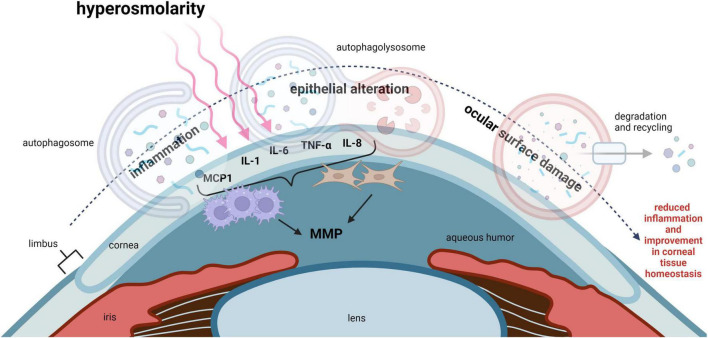
Autophagy regulates inflammation in DED and improves corneal epithelial homeostasis. Hyperosmolarity, desiccating stress and chronic mechanical irritation are common stressors that trigger inflammation in DED. This leads to ocular surface damage. IL-1, IL-6, IL-8, TNF-α, and MCP1, have all been shown to be elevated in DED. These pro-inflammatory cytokines function to recruit leukocytes such as macrophages to the cornea. Inflammation-induced ocular surface damage is associated with activation of MMPs, where MMP3 and MMP9 play an important role in degenerative tissue remodeling. Available evidence suggests that autophagy may act as a key regulator of inflammation and restore corneal epithelial homeostasis in DED. MCP1, monocyte chemoattractant protein-1; IL-1, interleukin 1; IL-6, interleukin 6; IL-8, interleukin 8; TNF-α, tumor necrosis factor alpha; MMP, matrix metalloproteinase.

Dry eye disease (DED) is a multifactorial disease that affects the ocular surface and is characterized by deficient aqueous tear production and/or an increase in tear evaporation. The instability of the precorneal tear film and resultant hyperosmolarity triggers inflammation, leading to tissue damage and neurosensory abnormalities (33). In both corneal epithelial cells exposed to hyperosmolar stress and in animal models of DED, an increase in oxidative stress has been well documented (34). It is well established that oxidative stress activates autophagy in many cell types (35). This is a key mechanism to maintain cell viability and prevent apoptosis. Thus, in the corneal epithelium, autophagy may function to protect against corneal epithelial tissue damage and maintain ocular surface homeostasis (Figure 3).

Several studies have investigated the role of autophagy in DED using the desiccating stress mouse model. In a study by Ma et al., the authors reported increased expression of LC3B in the corneal epithelium after 5, 10, and 20 days of desiccating stress based upon immunofluorescence and an increase in the LC3B II/I ratio by immunoblotting (36). Autophagosome accumulation was also noted. Autophagy was further increased in mouse corneas treated topically with LYN-1604, a molecule known to induce autophagy through activation of the unc-51-like kinase (ULK) and then blocked using 3-methyladenine (3-MA), which inhibits phosphoinositide 3-phosphokinase (PI3K) (36, 37). Interestingly, LYN-1604 appeared to improve tear production in mice with DED and decreased the inflammatory markers tumor necrosis factor-alpha (TNF-α), matrix metalloproteinase (MMP)-3, and MMP-9 (36). In contrast, the inhibition of autophagy worsened all parameters, consistent with greater disease pathology in DED.

Trehalose is another known inducer of autophagy with fewer cytotoxic effects in mammalian cells than other chemical inducers (38). A recent study investigated the effects of trehalose on primary cultured human corneal fibroblasts and SV-40 immortalized corneal epithelial cells exposed to desiccating or inflammatory stress (39). They further examined the effects of trehalose when topically applied to the human cornea in patients with DED. In their cell culture models, neither TNF-α nor desiccating stress induced autophagy. The simultaneous treatment of cells with TNF-α and trehalose increased autophagy, evidenced by an increase in the LC3 II/I ratio, but this did not occur when trehalose was used in conjunction with desiccating stress. While trehalose was effective in the reduction of TNF-α-induced inflammation, it only reduced monocyte chemotactic protein-1 (MCP-1) and had no effect on IL-6, IL-8, and MMP-9 in the desiccating stress model. Similarly, in human studies, the administration of trehalose topically to the eye reduced the ocular surface disease index score, indicating reduced symptomology, and reduced MMP9. Consistent with their desiccating stress findings, IL-6 and IL-8 were unchanged in tears following trehalose treatment for DED (39).

Chloroquine is an antimalarial drug that has been shown to block autophagy by inhibiting lysosomal fusion with the autophagosome (40). In their *in vitro* desiccating stress model, Shivakumar et al. similarly found that autophagy was upregulated in corneal epithelial cells, evidenced by an increase in LC3B and the lysosomal marker LAMP1 (41). This was associated with an increase in NF-κB activity. Interestingly, rather than activate autophagy, they blocked autophagy in corneal epithelial cells using chloroquine. While this was effective in reducing NF-κB, it had no effect on autophagy induced by desiccating stress.

Liu et al. used primary human corneal epithelial cells (HCECs) to assess the effects of hyperosmolar stress on pro-inflammatory mediators (42). These included TNF-α, IL-1b, IL-6, and IL-8. They found that there was an increase in all four pro-inflammatory cytokines. They also reported an increase in autophagy markers ULK1, ATG5, Beclin 1, and LC3. Paradoxically, treatment of cells exposed to hyperosmolar stress with rapamycin, a known inducer of autophagy, suppressed inflammation and increased cell viability, albeit this latter effect was small in magnitude. The authors concluded that while autophagy is increased in cells exposed to hyperosmolar stress, further activation through rapamycin attenuated inflammation and promoted cell survival, without the induction of autophagic cell death.

Consistent with these prior studies, Wang et al. also showed that LC3B-II was increased in a desiccating stress mouse model of DED (43). They further demonstrated this *in vitro* using immortalized SV40 human corneal epithelial cells and primary cultured corneal epithelial cells exposed to hyperosmolar stress. Using electron microscopy, their data suggested a potential increase in autophagosome formation. They further showed evidence of accumulation of p62 in both *in vitro* and *in vivo* models, while Beclin 1 and ATG5 were unchanged. Similar to the increase in LC3II and p62, there was a parallel increase in the DNA damage-inducible transcript 4 (DDIT4). Previous work has found that the upregulation of DDIT4 is associated with DED and increased ROS production (44). In support of this view, Wang et al. found that siRNA-mediated knockdown of DDIT4 in corneal epithelial cells showed decreased levels of ROS in response to hyperosmolar stress (43). To evaluate whether the DDIT4-ROS axis mediates autophagy during hyperosmolar stress, N-acetylcysteine (NAC) was used. The addition of NAC restored levels of LC3-II and p62 to normal levels in HCECs *in vitro*. Likewise, in the DED mouse model, NAC exerted a protective effect on the ocular surface. The authors concluded that DDIT4 may function as an endogenous regulator of autophagy and mediate ROS-induced autophagy in DED.

Lacritin is a multifactorial tear glycoprotein that is produced by the lacrimal and meibomian glands, in addition to the corneal and conjunctival epithelium (45). Lacritin is an epithelial mitogen. Consistent with this role, lacritin has been shown to be an important molecule in corneal wound healing (46). A deficiency of lacritin in tear fluid has also been associated with DED (46–49). In a separate study by Wang et al., cells co-treated with inflammatory cytokines and tears from non-DED patients demonstrated cytosolic localization of the transcription factor FOXO3 (45). In contrast to this, treatment of cornea epithelial cells with pro-inflammatory cytokines and tears from patients with DED revealed a nuclear localized FOXO3, suggesting an impairment in corneal epithelial homeostasis in the dry eye condition. Interestingly, co-treatment of cells incubated with tears from dry eyes and lacritin reversed the nuclear localization of FOXO3, sequestering it in the cytosol. The authors went on to show that the lacritin-dependent improvement in corneal epithelial homeostasis appeared to be dependent on the activation of autophagy. This effect was mediated by the acetylation of FOXO3, suggesting that it functions as a novel Atg101 ligand.

Melatonin-loaded polymer polyvinyl caprolactam–polyvinyl acetate–polyethylene glycol graft copolymer micelles (Mel-Mic) have similarly been reported to ameliorate hyperosmolarity-induced ocular surface damage by augmenting autophagic flux and mitophagy *via* PINK1 (50). In this study, treatment of corneal epithelial cells subject to hyperosmolar stress with melatonin or melatonin-loaded micelles (Mel-Mic) led to an enhancement in LC3B and Beclin 1 expression, while p62 levels were decreased (50). When they assessed mitophagy, they found that expression of both PINK1 and Parkin were increased by treatment with Mel-Mic, while translocase of outer membrane 20 (TOM20) was downregulated. Similarly, the topical administration of Mel-Mic in mice with DED attenuated corneal epithelial cell surface damage and restored tear production. There was also an increase in the number of conjunctival goblet cells. Similar to their *in vitro* findings, LC3B was increased in the cornea of dry eye mice treated with Mel-Mic and there was a corresponding decrease in the expression of TOM20 (50). Autophagosomes were also observed by transmission electron microscopy of corneal epithelial tissue treated with Mel-Mic, along with an increase in the expression of PINK1 and Parkin. Collectively, Mel-Mic administration showed an improvement in autophagic flux and mitophagy and this was associated with the alleviation of ocular surface damage in DED (50).

### 2.3. Limbal stem cells

Limbal stem cells are essential for the maintenance of the corneal epithelium. Deficiencies in the limbal stem compartment are associated with conjunctivalization of the corneal surface. Several studies have implicated autophagy as a critical mediator of the limbal stem cell compartment (51, 52). Specifically, autophagy has been shown to exert a protective effect on the limbal epithelium (51, 53). In Beclin 1 deficient (+/-) mice, RNA sequencing studies have shown a reduction in genes that drive proliferation. One of the most highly affected genes in this study was the PDZ-binding kinase (PBK). Importantly, siRNA knockdown of PBK in hTCEpi cells triggered cell cycle arrest (54). The authors speculated that the change in expression of PBK likely explains the 50% decrease in stem and early transient amplifying cells in Beclin 1 +/- mice.

H2A histone family member X (H2AX) expression was also much lower in the corneal epithelium of Beclin 1 +/- compared to wild type mice, suggesting the presence of an impaired stress response due to cells not being able to undergo proper DNA damage repair (54). Likewise, the activating transcription factor 3 (ATF3) was upregulated in the limbal stem cell compartment. When hTCEpi cells were interrogated after siRNA knockdown of ATF3, there was a significant increase in cell growth. Thus, increased expression of ATF3 in Beclin 1 +/- mice may attenuate epithelial renewal. This would also be consistent with the reported decrease in corneal epithelial wound healing.

miR-103/107 is a miRNA that is predominantly expressed in the basal cells of the limbal epithelium. A prior study has reported that miR-103/107 regulates genes that function in stem cell maintenance (52). Subsequent work by this same group explored the relationship between miR103/107 and autophagy. Using antagomirs to silence miR-103/107 and bafilomycin to block autophagosomal-lysosomal fusion, they found that late-stage autophagy was impaired. This occurred through an increase in protein kinase C (PKC) activity, phosphorylation of dynamin, and an attenuation in end stage autophagy. Since autophagy has been proposed as a potential regulator of the stem cell niche, this same group went on to further show that blocking autophagy by treating cells with bafilomycin decreased holoclone formation. Consistent with this, corneal epithelial wound healing was impaired in Beclin +/- deficient mice.

As discussed previously, the induction of autophagy has been linked to oxidative stress and ROS production (55). Thus, increased autophagy has been suggested to reduce oxidative stress and mitigate tissue damage (56). Given the crucial role for autophagy in the protection of limbal stem cells, Zhou et al. investigated the effects of H_2_O_2_ on murine corneal epithelial progenitor cells (TKE2) compared to mature murine corneal epithelial (MCE) cells (57). Importantly, the authors reported that TKE2 cells treated with H_2_O_2_ increased expression of superoxide dismutase (SOD) and glutathione S-transferase P (GSTP) and autophagy, while suppressing levels of ROS. In contrast, the opposite effects were seen in MCE cells. In addition, Beclin 1 was upregulated in TKE2 cells and downregulated in MCE cells. This paralleled changes in the ratio of LC3-II to LC3-I. Together, these data suggest that the TKE2 progenitor cells resist oxidative stress by enhancing autophagy, whereas oxidative stress inhibits autophagy in mature MCE cells.

### 2.4. Keratoconus

Keratoconus is a progressive ectatic corneal disease where stromal thinning leads to the formation of a cone due to the abnormal protrusion of the weakened cornea. This results in irregular astigmatism and visual compromise. Many of these patients require surgical intervention at some point in the disease process, whether it be corneal cross-linking to prevent further thinning or penetrating keratoplasty to restore vision. Increased levels of oxidative stress have been reported in keratoconic corneas. Due to the relationship between oxidative stress and autophagy, several studies have attempted to link the increase in oxidative stress with changes in autophagy in keratoconus.

Yildiz et al. (58) investigated PINK1-mediated mitophagy in human corneal epithelium collected from patients undergoing photorefractive keratectomy or corneal cross-linking for myopia or keratoconus, respectively (59). Evaluation of both mRNA and protein revealed a significant decrease in PINK1 in the epithelium of keratoconic patients. There were no changes in expression levels of ATG5, LC3B, or Beclin 1. In contrast to this work, Iqbal et al. (60) investigated LC3 expression using immunohistochemistry in 13 keratoconic corneas compared to controls and reported higher expression levels in three of the keratoconus samples (59). Finally, Shetty et al. (61) investigated the expression of autophagy markers in corneal epithelial tissue from 78 patients with keratoconus that were undergoing corneal cross-linking (62). They reported differential expression of LC3 and p62 in keratoconic tissue compared to controls, with a clear decrease in LC3 expression at all grades of severity. Collectively, these findings are somewhat equivocal and highlight the need for further studies to investigate the role of autophagy and mitophagy in keratoconus.

### 2.5. Fuchs endothelial corneal dystrophy

**FIGURE 4:**
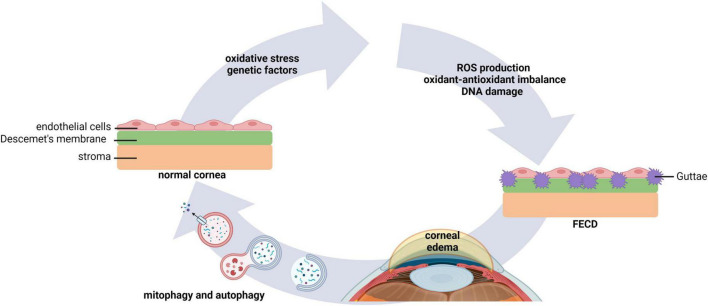
Autophagy and mitophagy play key roles in FECD. Environmental factors that lead to oxidative stress, as well as genetic factors, underlie morphological changes in corneal endothelial cells. This leads to an increase in ROS production, oxidant-antioxidant impairments, mitochondrial fragmentation, and DNA damage. Extracellular matrix excrescences form in Descemet’s membrane. Dysregulated pump function in the compromised corneal endothelium causes stromal edema and vision loss. Evidence suggests that modulation of mitophagy and autophagy may improve corneal homeostasis in FECD. FECD, Fuchs endothelial corneal dystrophy.

Fuchs endothelial corneal dystrophy (FECD) is one of the most common corneal endothelial dystrophies worldwide. FECD is characterized by a bilateral heterogeneous progressive degeneration in corneal endothelial cells (62, 63). The pathophysiology of FECD is marked by the loss of corneal endothelial cells and the presence of extracellular matrix excrescences in Descemet’s membrane, known as guttae. Guttae are encircled by cells forming rosette structures. This leads to endothelial dysfunction, corneal edema, and vision loss (64). Changes in the intracellular environment of corneal endothelial cells in FECD are associated with macromolecular alterations and senescence, where cellular stress stimulates ROS production, an imbalance in the oxidant-antioxidant response, and mitochondrial damage (Figure 4) (65–67). Given the abundance of mitochondria within the corneal endothelium, it is not surprising that mitochondrial dysfunction plays a central role in FECD (68).

Mitochondrial dynamics, fusion and fission, are associated with mitochondrial quality control in response to damaged mitochondria (65). Under conditions of cellular stress, unsalvageable mitochondria undergo fission and are then targeted for degradation by mitophagy. It is well-established that the loss of mitochondrial membrane potential is a key trigger for PINK/Parkin-mediated mitophagy (65, 69). One of the ubiquitination targets for Parkin is the fusion protein mitofusin 2 (MFN2). The degradation of MFN2 in turn promotes mitochondrial fragmentation, allowing for the removal of damaged mitochondria (65). Indeed, in corneal tissue collected from patients with FECD, there is a reduction in MFN2 expression (70, 71). This decrease in MFN2 is associated with a fission dominant morphology and mitochondrial fragmentation. This was further associated with a decrease in mitochondrial mass, and the activation of fission-induced mitophagy, evidenced by an increase in levels of LC3-II and the lysosome associated membrane protein 1 (LAMP1).

Based upon these findings, a subsequent study also evaluated mitochondrial changes and mitophagy in post-surgical FECD tissues compared to normal corneas (72). Here, the authors demonstrated excessive mitochondrial clearance through mitophagy in FECD. The association of PINK1, phosphorylated Parkin and autophagosome formation were shown in conjunction with down-regulation of dynamin related protein 1 (DRP1) (72). While DRP1 is another key protein that regulates mitochondrial fission, DRP1 is not necessarily required for mitophagy (73, 74). To complement their *ex vivo* tissue studies, the authors used menadione (2-methyl-1,4-naphthoquinone) to induce robust oxidative damage of nuclear and mitochondrial DNA in cultured human corneal endothelial cells (HCEnC)-21T cells. Importantly, Parkin-dependent mitophagy was found to be a key regulator in this system (72). Moreover, the ROS-dependent translocation of YFP-Parkin was observed in fragmented mitochondria, together with upregulation of phosphorylated Parkin, suggesting the induction of mitophagy in a PINK1-independent manner. Collectively, these data support that increased oxidative stress in corneal endothelial cells mediates mitophagy through Parkin-mediated mitochondrial fragmentation in FECD, while DRP1 and PINK1 are degraded during the process of degenerative cell loss in the corneal endothelium.

In addition to mitophagy, the role of autophagy in FECD has also been investigated. Since one of the known pathophysiological changes in FECD is the abnormal accumulation of collagen type VIII (COL8), investigators have been using two novel mouse models that over-express collagen 8, Col8a2L^450W/L450W^ and Col8a2Q^455K/Q455K^. In both models, there is an upregulation of LC3 and autophagy proteins ATG12-5 (75). This was associated with autophagosome formation in the 40-week-old mutant endothelium. Analysis of the expression of autophagy-related genes at 40 and 80 weeks showed increased expression of the DNA-damage regulated autophagy modulator (DRAM1) and the transmembrane protein 74 (TMEM74) in the corneal endothelium of Col8a2L^450W/L450W^ mice compared to the wild type control. The increased expression of DRAM1 and TMEM74 at 40 weeks were also observed in the corneal endothelium of Col8a2Q^455K/Q455K^ mice, along with the phosphoinositide-3-kinase regulatory subunit 4 (PIK3R4). At 80 weeks, increased gene expression was observed in the corneal endothelium of Col8a2Q^455K/Q455K^ mice. This included the eukaryotic translation initiation factor 2-alpha kinase 3 (EIF2AK3), the mechanistic target of rapamycin (mTOR), cathepsin S (CTSS), TMEM74, DRAM1, and tumor necrosis factor receptor superfamily member 6 (Fas), all of which were upregulated compared to the wild type control.

The solute carrying family 4 member 11 (SLC4A11) is a voltage regulated cotransporter important in mediating corneal endothelial cell function (76). A recent study evaluated the effect of SLC4A11 on mitochondria and autophagy in congenital hereditary endothelial dystrophy (CHED) (77). In that report, the authors used primary human corneal endothelial cells (pHCEnCs) with and without knockdown of SLC4A11 to compare the effect of reduced expression on the transcriptome, along with Slc4a11^–/–^ mouse corneal endothelial cells (Slc4a11^–/–^ MCEnCs). In both models, there was evidence of dysfunction in ion transport, metabolic alterations and mitochondria. Validation studies confirmed an increase in Ser15 phosphorylation of p53 in SLC4A11 knockdown and null cells compared to controls, indicating post-translational activation of p53 transcriptional activity (77). Similarly, there was an increase in the phosphorylation of AMPKα at Thr172, which mediates Ser15 (Ser18 for mouse) phosphorylation of p53, but no changes were detected in phosphorylation of Ser182 of AMPKβ1. Collectively, this study provided evidence to support that SLC4A11 deficiency leads to corneal endothelial dysfunction through decreased production of ATP, resulting in the activation of adenosine monophosphate-activated protein kinase (AMPK) and it downstream targets p53 and ULK (77).

Given the protective role of lithium in cells exposed to increased levels of oxidative and endoplasmic reticulum (ER) stress, Kim et al. tested the effects of lithium on corneal endothelial cell survival *in vitro* and in their collagen knock in mouse model, Col8a2Q^455K/Q455K^ (78). Here, the authors show that in cells treated with either thapsigargin, an inducer of ER stress, or H_2_O_2_ to induce oxidative stress, lithium increased cell viability and autophagy. In their mouse model, the topical administration of lithium enhanced endothelial cell density (78). Electron microscopy revealed the presence of enlarged autophagosomes containing degraded mitochondria in the endothelium of lithium treated corneas. Lastly, lithium upregulated autophagy related proteins ATG5-12, p62, TMEM74, TMEM166, and TM9SF1. This suggests that lithium helped combat increased cellular stress through the induction of autophagy. Further investigations are needed to determine whether lithium or other autophagy modulating drugs will be beneficial as potential therapeutics for FECD and other endothelial dystrophies.

### 2.6. Antioxidant defenses

#### 2.6.1. Oxidant-antioxidant responses in the cornea

Oxidative stress plays an essential role in the pathogenesis of many human diseases (79). Oxidative stress occurs when intracellular levels of different free radical species become elevated, leading to cellular damage. ROS is the most studied free radical and is known to play an important role in stress-related ocular conditions (80–82). In efforts to combat oxidative stress, antioxidants are produced. Antioxidants function to prevent, block and/or repair cellular and tissue damage (79). The most common antioxidant molecules in the context of intracellular activity includes superoxide dismutase, which is responsible for the conversion of superoxide anions (O^2–^) into H_2_O_2_ and O_2_, and catalase and glutathione peroxidase, which convert H_2_O_2_ into H_2_O and O_2_ (83). Thioredoxin and peroxiredoxin exhibit antioxidant activity *via* regulation of the dithiol/disulfide balance (84). Lastly, glutathione S-transferase can act through a Se-independent glutathione-peroxidase to reduce lipid hydroperoxides (85). In addition, non-enzymatic antioxidants also function to reduce oxidative stress. Non-enzymatic antioxidants include metal-binding proteins, ascorbic acid, vitamin E, uric acid, and glutathione (86).

The ocular surface is constantly exposed to stressors derived from the air and the environment, including pollutants, microbes, radiation, and desiccation. These stressors induce the production of free radicals, leading to increased oxidative stress-induced damage of the corneal and conjunctival epithelium. Trehalose is a disaccharide that has been shown to function as an antioxidant through the reduction of ROS (87). As we previously described in section “2.2. Dry eye disease,” trehalose is also a known inducer of autophagy (88). Indeed, evidence suggests that trehalose enhances autophagy through AMPK signaling (89, 90). Despite the ability of trehalose to mediate autophagy, its antioxidant activity appears to be autophagy independent and instead is mediated by phosphorylation of the autophagy receptor p62. Here, trehalose-mediated phosphorylation of p62 drives nuclear translocation of the nuclear factor (erythroid-derived 2)-like 2 (NRF2) protein, an integral regulator of antioxidant signaling. Similar to trehalose, melatonin has also been shown to function as both an antioxidant and an inducer of autophagy with beneficial effects on the ocular surface (50, 91–93).

NRF2 signaling and autophagy were also evaluated in murine corneal epithelial cells. In their study, Zhou et al. used both mature and progenitor corneal epithelial cells subject to H_2_O_2_-induced oxidative stress (57). They found that murine corneal epithelial progenitor cells exhibited a potent antioxidant phenotype that was mediated by NRF2 (57). This was associated with an increase in autophagic flux. In contrast to this, robust oxidative stress was induced in mature murine corneal epithelial cells and this was associated with an inhibition of autophagy. These findings suggest a potential interplay between cellular redox state and autophagy. However, much work remains to determine how the different antioxidants may interact and/or mediate autophagy in the cornea.

## 3. Infectious keratitis

### 3.1. Viral keratitis

Viral keratitis is a major cause of corneal blindness in the United States and western countries (59). The most common virus associated with keratitis and subsequent corneal scarring due to repeat infection is herpes simplex virus (HSV-1 and HSV-2) (94). Other common viruses that infect the cornea include varicella-zoster virus (VZV) or herpes zoster virus, cytomegalovirus (CMV), and Epstein-Barr virus (EBV). In rare cases, rubeola virus has also been linked with viral keratitis (95, 96). Little evidence is present in the literature on the role of the host autophagy response in the pathogenesis of viral keratitis. Past studies have highlighted the importance of autophagy in immune cells however, and a regulatory role for innate immune sensors (97–99).

Autophagy has been shown to function as a mechanism that affords virus survival inside host cells. In a Statens Seruminstitut rabbit corneal (SIRC) epithelial cell model, both HSV-1 and HSV-2 infection were shown to be capable of inducing host cell autophagy (98). This was demonstrated by confirmation of LC3 lipidation and an increase in autophagosomes. The inhibition of autophagy using the autophagy inhibitor bafilomycin decreased levels of the HSV envelope glycoprotein D, reduced viral replication, and promoted corneal epithelial cell apoptosis, suggesting that the induction of autophagy and prevention of apoptosis is important for viral replication. In contrast to this, Ames et al. showed that in the cornea, activation of the host autophagy receptor optineurin (OPTN) aids in the degradation of HSV-1 infectious particles and regulates the adaptive immune response (100). Using the cornea scarification model, they further showed that two thirds of OPTN^–/–^ mice infected with HSV succumbed to death. Likewise, Yakoub et al. found that the induction of autophagy by starvation or pharmacological activation significantly downregulated HSV-1 replication in the corneal epithelium, retinal ganglion cells and embryonic fibroblasts (101).

Both innate and adaptive immune responses are critical to control HSV-1 infection. Of these, sustained activation of pro-inflammatory CD4+ T cells leads to the formation of a sight threatening inflammatory lesion in HSV stromal keratitis (102). Jiang et al. investigated the upstream pathways responsible for mediating CD4+ T cell activation during HSV-1 infection (99). In that study, they used mice with dendritic cells that lack ATG5, resulting in dendritic cells that are unable to undergo autophagy. They found that these mice exhibited a normal innate immune response but impaired Th cell immunity. While the absence of an adaptive immune response had no effect on overall viral load, it did decrease CD4+ T cell activation and pathological corneal inflammation.

The cytosolic DNA sensor signaling pathway, stimulator of interferon genes (STING), has also been shown to be critical in preventing HSV-1 mediated mortality during intravenous infection in mice (103). However, while STING^–/–^ mice inoculated with HSV *via* the eye demonstrated worsening periocular disease and higher viral titers compared to the wild type control, they did not experience the same increase in mortality that was evident during intravenous infection. They concluded that STING is important for mediating viral loads in the cornea, but not in mortality when inoculated *via* the cornea. The authors further used a low virulence strain of HSV that did not antagonize host cell autophagy. In mice with an active STING response, the mutant HSV strain failed to induce death. In contrast to this, in STING^–/–^ mice, the effect of the mutant strain on animal death was identical to that induced by a second mutant that did not antagonize host cell mitophagy. Thus, the authors concluded that activation of STING-dependent autophagy is an essential antiviral response in the central nervous system (97).

While autophagy has been shown to play a role in mediating HSV infection, it is unclear whether mitophagy is involved. In non-ocular tissue, prior work has shown that both HSV-1 and HSV-2 infection in keratinocytes promote mitochondrial dysfunction (104). Using a dermal keratinocyte cell line (HaCaT cells), changes in mitochondrial localization and morphology have been observed during the initial stages of HSV infection (104). In this work it was shown that infection triggered an increase in mitochondrial fission. This led to robust mitochondrial fragmentation and was associated with a shift in mitochondria toward a perinuclear localization. Similar to this, HSV-1 and HSV-2 infection in primary neurons *in vitro* also increased mitochondrial fission and decreased mitochondrial membrane potential (105). This was associated with an increase in ROS production. While an increase in mitochondrial fission and loss of mitochondrial membrane potential may promote PINK1-mediated mitophagy, no studies to date have established a link between PINK1-mediated mitophagy and HSV infection.

Like HSV, VZV is also an alpha herpesvirus that invades the nervous system. Primary infection in patients seronegative for VZV results in varicella (chickenpox). During this stage, virus particles in skin lesions undergo retrograde migration to invade either the dorsal root or trigeminal ganglia, where it remains latent for years. Reactivation of the virus, usually seen in the immune compromised or the elderly, triggers a localized mid-line respecting vesicular rash on the face known as shingles. In the United States, it is estimated that up to 20% of these cases can involve the eye and adnexa, a condition known as herpes zoster ophthalmicus (HZO) (106).

Several studies have demonstrated a pro-viral role for the induction of autophagy during VZV infection (107–112). This has included work showing an upregulation of autophagy during VZV infection in various models including human fibroblast cell lines, primary cells, keratinocytes, and melanocytes (108–112). In contrast to this, in a fetal lung fibroblast cell line, VZV was shown to induce autophagy, as shown by an increase in LC3 lipidation, but attenuated autophagic flux at later stages of the pathway (113). Amino acid starvation and the chemical activation of autophagy reduced viral titers, but when treated with bafilomycin, the block in autophagy increased viral titers. Taken together, this work suggests that viral induction of autophagy to induce vesicle formation without lysosomal degradation is pro-viral. In a subsequent study, it was shown that bafilomycin also functions during viral infection to block secondary envelopment due to disruption of the virus assembly complex (114). The authors speculate that these effects were due in part to the effects of bafilomycin on the Golgi complex, however, further study is necessary to confirm these findings.

In addition to autophagy, VZV infection has also been shown to alter mitochondrial morphology. Using human fetal lung fibroblasts, the authors reported on the diffusion of the immediate early p63 (IE63) protein into mitochondria during infection (115). Moreover, throughout the duration of the study, mitochondria underwent significant morphological changes, including swelling and fragmentation. Despite a report of mitochondrial abnormalities, no studies to date have evaluated the role of mitophagy in VZV infection. Moreover, all available data on the role of autophagy in VZV has been acquired using non-ocular models (116). For an in depth review on what is known on the relationship between autophagy and VZV infection, the reader is directed to the review by Heinz et al. (116).

Unlike HSV and VZV, CMV is a betaherpesvirus that causes asymptomatic infection in otherwise healthy individuals. In an immune compromised patient, dissemination of CMV occurs by invasion of CD34+ myeloid progenitor cells. Multi-organ involvement can follow as a result of viral shedding to different organs *via* macrophages and dendritic cells. CMV keratitis is an uncommon but known ocular manifestation causing epithelitis, stromal keratitis, and endotheliitis (59). Of relevance to this review, CMV is known to modulate autophagy in human fibroblasts and to block autophagic flux through the insulin receptor substrate (IRS1) and the tegument protein (TRS1) by degrading autophagosome cargo (117, 118).

During the initial stages of CMV infection of human skin fibroblasts and astrocytoma cells, an increase in mitochondrial biogenesis and oxidative phosphorylation has also been reported (119–121). This is due to an increased energy demand for fatty acids to facilitate formation of the viral membrane. A more recent study has shown that the viral protein pUL13 targets mitochondrial cristae architecture to promote mitochondrial respiration during infection (122). In contrast to this, the immediate early viral protein pUL37 × 1 has been shown to induce mitochondrial fragmentation in human fibroblasts (123–125). Similar to VZV, the effects of CMV on the mitochondria have been described, but the role of mitophagy is unknown. In contrast to this, studies have demonstrated that the induction of autophagy during CMV infection improves the ability of the virus to replicate (126, 127). It remains unknown whether the autophagic response during CMV infection is cell type specific and if these findings are relevant to the eye.

Epstein-Barr virus is a gammaherpesviruses that causes infectious mononucleosis (IM). The worldwide prevalence of EBV is estimated to be 90% (128). Also known as glandular fever, IM causes fever, lymphadenopathy, and pharyngitis. Chronic EBV infection is also responsible for ocular manifestations, including dry eye and infectious keratitis (129). Studies using several lymphoma and embryonic kidney cell lines have shown that EBV highjacks the autophagic degradation pathway to survive intracellularly and promote viral replication (130). This occurs following the shift from latent infection to the induction of lytic replication. This is regulated *via* the extracellular signal-regulated protein kinase (ERK) 1/2, protein kinase c (PKC) theta-p38 mitogen activated protein kinase (MAPK) axis or c-Jun N-terminal kinase (JNK) 1/2 pathways (131–133). EBV viral proteins including latent membrane protein-1 (LMP-1), LMP-2A and the EBV nuclear protein, EBNA-3C, are known to promote autophagy (134–136). In the absence efficient autophagosome production or the inhibition of autophagy, EBV has also been shown to recruit autophagy proteins ATG8-LC3 to its envelope in the cytosol to promote efficient envelope formation during the lytic cycle (137).

Autophagy does contribute to the anti-EBV immune response by regulating type I interferon (IFN) producing plasmacytoid dendritic cells and ROS producing monocytes (138, 139). Moreover, in lymphoma cells, autophagy is induced during the early stages of EBV infection in attempts to clear the virus, but is later attenuated by the virus during the lytic phase to promote survival (140). This was confirmed by demonstrating that the inhibition of autophagy during either the early or late phase of infection resulted in enhanced viral replication. Despite the body of work investigating the relationship between EBV and autophagy, the role of autophagy in the context of EBV keratitis is unexplored and there are no published studies on the role of mitophagy in EBV infection.

### 3.2. Bacterial keratitis

Bacterial invasion of the cornea can lead to sight threatening infectious keratitis. The most common risk factors for infectious keratitis include contact lens wear, trauma, and immunosuppression (141–143). In the case of contact lens-related infectious keratitis, *Pseudomonas aeruginosa* is the most commonly identified pathogen in culture proven cases (144). Other common bacteria include *Staphylococcus aureus*, *Streptococcus pneumoniae*, *Stenotrophomonas maltophilia*, *Enterobacteriaceae*, *Klebsiella* spp., *Serratia* spp., and *Proteus* spp., among others (145). Using an LC3-GFP reporter, Brothers et al. demonstrated that soluble proteins from several bacteria including *S. aureus*, *Serratia marcescens*, *Escherichia coli*, methicillin sensitive *S. aureus* (MSSA), and methicillin resistant *S. aureus* (MRSA) strains were capable of activating autophagy in a human corneal limbal epithelial cell line (146).

In contrast to this, *Achromobacter xylosoxidans, Klebsiella pneumoniae, Acinetobacter baumannii*, *Enterococcus faecalis*, *S. maltophilia*, and *Moraxella* spp. did not exert the same effect (146). The authors also demonstrated a greater amount of autophagy in MRSA compared to MSSA strains. Collectively, this study highlighted that a diverse array of bacterial pathogens are able to regulate host autophagy. In addition, host response varied with respect to the bacterial strain, with *S. marcescens* exhibiting the strongest response (146). Another study by the same group showed that *S. marcescens* mutants that lack function of *eepR* and gumB genes, both of which regulate multiple heat-stable secondary metabolites, were unable to promote autophagy in HCLE cells (147). Since targets of *eepR* and gumB include genes involved in the synthesis of prodigiosin (pigD), the authors tested a strain of *S. marcescens* with a mutation in pigD. Here they found that loss of the pigD gene abrogated autophagy.

In a different corneal epithelial cell line, Monankumar et al. reported on the ability of several different isolates of *P. aeruginosa* to induce autophagy (148). They found that the virulence signature played a key role. While the Type III secretion system (T3SS) promoted autophagy, T3SS mutants failed to induce autophagy. This was associated with the downregulation of Beclin 1. Treatment of cells with the autophagy inhibitors chloroquine and 3-methyl adenine increased intracellular bacterial load, while the use of Earle’s balanced salt solution to induce nutrient deprivation autophagy or rapamycin significantly reduced bacterial load in T3SS positive strains. Together, these findings demonstrate that autophagy is utilized by the host to help clear intracellular *Pseudomonas* from corneal epithelial cells during infection.

In non-corneal cell types, *Pseudomonas*’ type IV secretory system PGAP-1-like effector activates autophagy in HeLa cells *via* the ER stress response (149). The p21-activated kinase 1(PAK1)/Akt/mTOR pathway is involved in autophagy regulation during *Pseudomonas* infection in lung epithelium (150). *Pseudomonas* has also been shown to activate autophagy in alveolar macrophages and mast cells (151, 152). Autophagy has also been shown to prime neutrophils during bacterial sepsis (153). Neutrophils and macrophages are important players in the innate immune response during *Pseudomonas* keratitis (154). However, the role of immune cell autophagy in the context of ocular bacterial keratitis has not yet been explored. Further studies are required to identify the signaling pathways involved in autophagy regulation during PA keratitis.

*Pseudomonas* has been shown to induce mitochondrial damage in macrophages (155). Importantly, damaged mitochondrial DNA triggers activation of inflammasomes. This process is downregulated by autophagy (155). Mitophagy has been reported to be protective during PA infection by dampening the innate immune response during pathogen exposure. Pyoverdin, an iron-binding siderophore toxin from *Pseudomonas* alters iron and mitochondrial homeostasis to trigger mitophagy in *Caenorhabditis elegans* (156). Another study demonstrated the functional role of miRNA miR-302/367 in triggering mitophagy during PA infection. In this work, the upregulation of miR-302/367 promoted mitophagy in macrophages in efforts to clear the pathogen (157). PA’s type III secretion system exoenzyme effector ExoU has also been shown to induce mitochondrial dysfunction in murine macrophages.

*Streptococcus pneumoniae* keratitis is common among patients with other ocular diseases and following surgery or trauma to the eye (158, 159). In non-ocular systems, *S. pneumoniae* is known to exploit the host autophagic response for intracellular survival through choline-binding proteins (160). Studies have also identified autophagic regulation of neutrophils and macrophages during *S. pneumoniae* infection (161–163). At the level of the mitochondria, *S. pneumoniae* infection induces mitochondrial fission and mtROS production in macrophages (164). Similarly, PINK1 and Parkin-mediated mitophagy have been shown to prevent *Streptococcus* infection-induced lung damage by downregulating inflammasome activation and promoting apoptosis (165). The accumulation of PINK1 is known to occur in response to mitochondrial depolarization. However, studies using an *in vivo* mouse model have shown that despite the accumulation of PINK1 during *Streptococcus* infection, PINK1 was not associated with the outer mitochondrial membrane and therefore did not stimulate mitophagy. Thus, bacterial invasion was associated with frank mitochondrial damage (166).

### 3.3. Fungal keratitis

Fungal keratitis is a rare disease in western countries, but predominates in tropical and subtropical regions of the world. The prevalence may vary with respect to the regional climate and urbanization; however, *Fusarium*, *Aspergillus*, and *Candida* species are the most common fungal pathogens (167). Fungal keratitis can result in severe corneal ulceration and evisceration or enucleation of the globe. Risk factors for fungal keratitis include ocular trauma, injury, contact lens wear, corticosteroids use, and immunosuppression (168–170). Recent studies have highlighted a role for miRNAs in the regulation of autophagy in *Fusarium* mediated keratitis (171–173). Using a mouse model of *Fusarium solani* keratitis, Guo et al. demonstrated changes in the LC3II/LC3I ratio, along with loss of ATG5 compared to control and the accumulation of p62, suggesting an impairment of autophagic flux (172). This correlated with increased expression of miRNA miR-665-3P. Subsequent *in vitro* and *in vivo* blocking experiments using an antagomir targeting miRNA miR-665-3P were associated with an increase in autophagic flux. This increase corresponded to a decrease in corneal inflammation.

Work by this same group has also shown a regulatory role for miRNA miR-129-5p in negatively regulating autophagy in *F. solani* infected mouse corneal stromal cells (171). Here they demonstrated that subconjunctival injection of a miR-129-5p antagomir increased autophagy protein expression for LC3B and Beclin 1. They further showed that ATG14 was a direct target for miR-129-5p (171). In a third study, the same group again found impaired autophagic flux followed by upregulated miR-223-3p expression in their mouse model of *F. solani* infection (173). Here they found that miR-223-3p was found to negatively regulate the autophagy protein ATG16L1. Similar to their prior work, the inhibition of miR-223-3p increased autophagy while decreasing inflammation to promote healing.

In contrast to *F. solani*, *Aspergillus fumigatus* was found to induce autophagosome formation in both human and mouse corneas (174). Treatment with autophagy inhibitors further aggravated disease severity, while the induction of autophagy decreased severity. This occurred through the regulation of polymorphonuclear cell recruitment and differentiation and balancing of the pro-inflammatory to anti-inflammatory cytokine ratio (174). A subsequent study investigating *A. fumigatus* also found that infection promotes autophagic flux in corneal epithelial cells *in vitro* and in the corneal epithelium *in vivo*. They further showed that the induction of autophagy is *via* the cGAS-STING innate immune pathway (175). *In vivo* studies with *A. fumigatus* sensitive C57BL/6 and resistant BALB/c mice have further shown that expression of the autophagy markers LAMP-1, Beclin 1, and LC3 correlated with disease severity (176). The non-canonical autophagy protein, cell cycle proliferation gene 1 (CCPG1), has also recently been implicated in the pathogenesis of *A. fumigatus* infection (177).

Reports on *Candida albicans* infection have yielded differing results arguing in support or against the role of autophagy in the host response to non-ocular infection. Using autophagy defective ATG7^–/–^ mice, there were no differences reported in the susceptibility to fungal infection (178). Modulating autophagy had no effect on immune cell function and nucleotide polymorphisms in autophagy genes failed to correlate with cytokine production in Candidemia patients or healthy controls. However, more recent studies demonstrate the positive regulation of autophagy in the pathogenesis of *C. albicans* (179–182). The fungal pattern recognition receptor Dectin-1 has been shown to mediate LC3 recruitment, thereby increasing fungicidal activity in macrophages during *C. albicans* infection (179). In vaginal epithelium, autophagy has been shown to be essential for survival during fungal infection. This conclusion was supported by work showing that ATG5 gene knockdown increased epithelial cell death. Other studies have suggested that autophagy may function as a defense mechanism during vaginal *C. albicans* infection. Future work evaluating the role of *C. albicans* in a corneal infection model will help us to better understand the regulatory role of autophagy in host defense. To date, there have been no studies investigating the host mitophagy response during infection with fungal pathogens.

### 3.4. Protozoan keratitis

Protozoa that are known to cause ocular surface infections include *Acanthamoeba* (AC), *Toxoplasma*, and *Leishmania* (183, 184). While protozoan infections overall are rare, *Acanthamoeba* is the most common pathogen and is typically associated with contact lens wear. During *Acanthamoeba* infection, the protozoa is introduced to the eye *via* the contact lens. Inoculation of the lens occurs from exposure to water. Corneal trauma followed by exposure to contaminated water, soil, or agricultural work can also lead to the development of *Acanthamoeba* keratitis (183). One of the major barriers to treatment of *Acanthamoeba* keratitis is the ability of the cyst to resist killing. Autophagy has been shown to play a key role in the encystation of the amoeba, conferring protection to the pathogen (185–188). This includes autophagosomal degradation of mitochondria (189). Indeed, studies have identified several autophagy proteins that are involved in mediating encystment. These include ATG8, ATG8 isoform, ATG3, and ATG16L (185–188). Interestingly, the autophagy inhibitors 3-methyladenine (3MA) and wortmannin have been shown to be effective in reducing encystation (190). Contact lens multipurpose care solutions, which are ineffective against *Acanthamoeba* cysts, combined with the autophagy inhibitors 3-methyladenine or chloroquine have been shown to reduce the adhesion rate of *Acanthamoeba* to contact lens surfaces (191, 192). Together, these studies provide strong evidence that autophagy is a critical player in the pathogenesis of *Acanthamoeba* keratitis.

*Toxoplasma gondii* is a known etiological agent for retinitis. In some cases, *T. gondii* is also associated with keratitis. In contrast to *Acanthamoeba*, autophagy functions to clear the pathogen, making it an important component in the host defense against *T. gondii* ocular infection (193, 194). During infection, *T. gondii* forms special parasitophorous vacuoles that allow them to evade the host immune system and safely replicate intracellularly. This is associated with increased superoxide levels and robust mitochondrial dysfunction (195). The adaptive immune system has been shown to play a key role in the induction of autophagy and the subsequent degradation of these vacuoles. The CD40–CD154 ligand interaction is one mechanism whereby the infected cell upregulates Beclin 1, leading to autophagocytic removal of the pathogen (193). *T. gondii* however, has developed mechanisms for inhibiting autophagy and lysosomal degradation. In a mouse model of ocular *Toxoplasmosis* infection, the pathogen has been shown to negatively regulate autophagy by inducing prolonged epidermal growth factor (EGFR) signaling by releasing micronemal proteins with EGF domains (194). This leads to activation of PKCa/b. The use of tyrosine kinase inhibitors was further shown to block EGFR signaling, leading to Beclin 1-mediated autophagic killing. This was confirmed in a separate study that also showed that the inhibition of EGFR signaling or infection by parasites that did not express a key micronemal protein led to an increase in parasite killing through autophagosome/lysosome fusion (196).

Leishmaniasis is a tropical disease that is transmitted by sand flies after consumption of a blood meal (197). Ocular manifestations of Leishmaniasis, known as Kala-Azar, mainly affects the retina (198). In cutaneous Leishmaniasis caused by bite near the eyelid, blepharoconjunctivitis, dacryocystitis, and even keratitis can develop (199, 200). In the case of corneal disease, if not adequately treated, infection can lead to perforation. Several *in vitro* and *in vivo* studies have shown that autophagy is important in the pathogenesis of *Leishmania* infection (201). In chronic kidney disease, the accumulation of autophagy proteins Atg7 and LC3 has been positively correlated with *Leishmania infantum* disease severity suggesting that host autophagy favors pathogen survival (202). *In vitro* studies of *Leishmania amazonensis* have shown that the induction of autophagy in macrophages is associated with a high infection index *in vitro* (203). Similarly, the induction of autophagy in Balb/c mice has been shown to increase the number of infected macrophages and the overall parasite burden *in vivo* (204).

**FIGURE 5:**
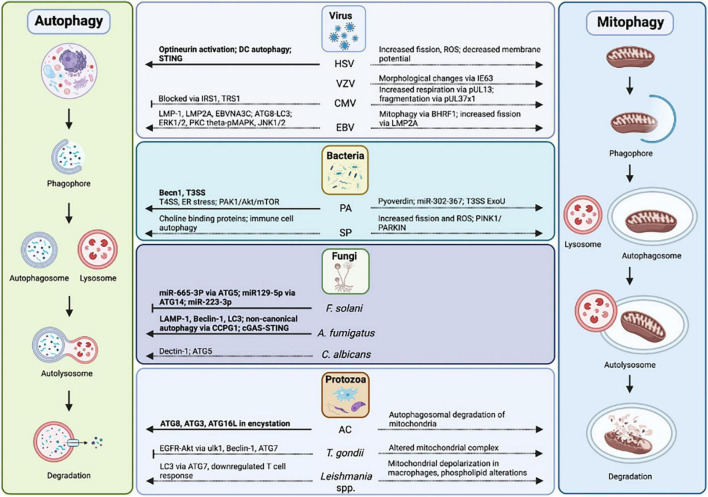
Autophagy and mitophagy in infectious keratitis. Autophagy has been studied in HSV, *P. aeruginosa*, *F. solani, A. fumigatus*, and *Acanthamoeba*-mediated keratitis. Autophagy proteins, pathways, mitophagy, and mitochondrial changes in infectious keratitis (in bold) compared to non-ocular infections.

Infection of human bone marrow by the *Leishmania donovani* complex has also been shown to induce autophagy (205). This effect was abrogated by treatment with amphotericin B. In human monocyte-derived macrophages, the induction of autophagy using rapamycin decreased T cell proliferation, increasing parasite survival (206). In a similar study, intravenous infection of Balb/c mice with *L. donovani* led to the production of CD4+ and CD8+ T cells that expressed the programmed death ligand-1 (PDL-1). The inhibition of PDL-1 blocked autophagy. This enhanced T-cell protective responses and led to a reduction in the parasitic load (59). Finally, infection of bone marrow derived macrophages obtained from Balb/c mice has shown distinct RNA and protein changes during early and late stages of infection (207). This data contradicted other work by showing that autophagy induced by *Leishmania* promastigotes was capable of clearing the parasite. This was confirmed by knocking down expression of either Atg5 and ubiquitin, both of which blunted autophagy while increasing the overall parasitic load. A summary of the organisms and their associated autophagic pathways are detailed in Figure 5.

## 4. Discussion

Autophagy has been investigated in the context of corneal diseases, infectious and non-infectious. In diabetes, evidence suggests there is a suppression of autophagy and mitophagy in the corneal epithelium and endothelium, in addition to trigeminal neurons. Based upon the few studies that are available, the induction of autophagy may be beneficial in resurfacing wounds in the diabetic cornea. While few studies have been performed and much work remains, the reports to date are consistent and suggestive of a modulatory role for autophagy in diabetes. The role of autophagy in dry eye disease is much less clear. The autophagic response is variable. This is likely due to differences in cell lines and animal models, types, and duration of stress. In addition, the effects of autophagy in ocular surface immune cells remains an area of heightened interest. Likewise, while Substance P, calcitonin gene related peptide, and nerve growth factor have been shown to modulate autophagy in non-ocular tissues, the role of these factors in autophagy in the corneal epithelium and corneal nerves is undefined (208–212). Moving forward, it is essential that appropriate controls are used and proper protein normalization is performed to substantiate and confirm some of these early findings.

In the limbal stem cell compartment, studies to date indicate that autophagy is protective in terms of proliferation and the DNA damage response. For much of this work, the inclusion of the Beclin 1 knockout mouse has been invaluable. In keratoconus, data in current reports is equivocal. Most reported findings are based upon epithelium harvested from the human cornea during corneal cross linking, in the case of keratoconic patients, and photorefractive keratectomy, for normal controls. There is some work that suggests that mitophagy may be downregulated in the epithelium from keratoconic eyes, but additional studies are needed. In contrast to this, the strongest data confirming an important role for mitophagy is in the corneal endothelium. This cellular monolayer plays a critical role in corneal transparency through it is ability to extrude water that is taken up by proteoglycans in the stroma. Given the high number of mitochondria that are required to maintain the pump, it is not surprising that defects in mitophagy, an essential mitochondrial quality control mechanism, have been found. Whether manipulation of these pathways may promote corneal endothelial health in patients with endothelial dystrophies remains to be proven, but is certainly an area of keen interest.

In terms of infectious disease in the cornea, huge gaps in our knowledge abound. In terms of viral infection, evidence exists to suggest that autophagy functions in a virus and cell type dependent manner. Autophagy has also been shown to affect the innate and adaptive immune systems. In the case of bacterial infection, some pathogens have been shown to induce autophagy during infection, while others have not. The strength of the response is further varied by bacteria strain and virulence. This is well exemplified by differences between methicillin resistant and methicillin sensitive *S. aureus*. Whether the induction of autophagy confers a gain or loss to the host remains to be determined. Mitophagy has also been shown to play a major role in the immune cell’s response. Overall, however, there is a paucity of data to define a clear role for autophagy and mitophagy in bacterial keratitis.

The same is true for fungal disease. Available data suggests that miRNAs, Beclin 1, and the cGAS-STING pathways may play key roles in mediating autophagy during fungal keratitis. To date however, not one study addresses mitophagy, despite aberrant mitochondrial damage that has been found during fungal infection in non-ocular tissues. In terms of protozoan infections, most striking and of high relevance to the cornea, is the ability of autophagy to promote *Acanthamoeba* encystation. As this is a particularly difficult pathogen to eradicate, the ability to block encystation and promote pathogen killing could represent a major advantageous step forward in our ability to treat these infections clinically.

Collectively, this work highlights the major gaps in knowledge that exist with respect to autophagy and mitophagy in non-infectious and infectious corneal diseases. While emerging data implicates a role for these pathways in corneal homeostasis, much work is needed to establish and refine the key players that are disturbed in corneal diseases and whether therapeutics designed to target these pathways may be beneficial.

## Author contributions

RA, JS, and DR wrote the manuscript. All authors contributed to the article and approved the submitted version.
